# Superparamagnetic Hyperthermia Study with Cobalt Ferrite Nanoparticles Covered with γ-Cyclodextrins by Computer Simulation for Application in Alternative Cancer Therapy

**DOI:** 10.3390/ijms23084350

**Published:** 2022-04-14

**Authors:** Isabela Simona Caizer, Costica Caizer

**Affiliations:** 1 Department of Plastic and Reconstructive Surgery, Faculty of Medicine, “Victor Babes” University of Medicine and Pharmacy of Timisoara, 300041 Timisoara, Romania; isabela.caizer@umft.ro; 2 Department of Clinical Practical Skills, Faculty of Medicine, “Victor Babes” University of Medicine and Pharmacy of Timisoara, 300041 Timisoara, Romania; 3 Department of Physics, Faculty of Physics, West University of Timisoara, 300223 Timisoara, Romania

**Keywords:** superparamagnetic hyperthermia, specific loss power, cobalt ferrite nanoparticles, gamma-cyclodextrins, magnetic relaxation, alternative cancer therapy

## Abstract

In this paper, we present a study by computer simulation on superparamagnetic hyperthermia with CoFe_2_O_4_ ferrimagnetic nanoparticles coated with biocompatible gamma-cyclodextrins (γ-CDs) to be used in alternative cancer therapy with increased efficacy and non-toxicity. The specific loss power that leads to the heating of nanoparticles in superparamagnetic hyperthermia using CoFe_2_O_4_–γ-CDs was analyzed in detail depending on the size of the nanoparticles, the thickness of the γ-CDs layer on the nanoparticle surface, the amplitude and frequency of the alternating magnetic field, and the packing fraction of nanoparticles, in order to find the proper conditions in which the specific loss power is maximal. We found that the maximum specific loss power was determined by the Brown magnetic relaxation processes, and the maximum power obtained was significantly higher than that which would be obtained by the Néel relaxation processes under the same conditions. Moreover, increasing the amplitude of the magnetic field led to a significant decrease in the optimal diameter at which the maximum specific loss power is obtained (e.g., for 500 kHz frequency the optimal diameter decreased from 13.6 nm to 9.8 nm when the field increased from 10 kA/m to 50 kA/m), constituting a major advantage in magnetic hyperthermia for its optimization, in contrast to the known results in the absence of cyclodextrins from the surface of immobilized nanoparticles of CoFe_2_O_4_, where the optimal diameter remained practically unchanged at ~6.2 nm.

## 1. Introduction

In cancer therapy, it has now become more and more necessary to find new alternative methods to the conventional ones (chemo- and radiotherapy) such as magnetic hyperthermia [1,2,3,4,5], photothermal therapy [6,7,8,9], or theranostic [10,11,12,13], which would lead to an increase in efficacy, but especially to a reduction in toxicity and side effects on the human body such as those caused by the classic chemotherapy and/or radiation therapy, techniques currently used in the treatment of cancer. Magnetic hyperthermia or recently superparamagnetic [13,14,15] is one of the most promising alternative methods in this issue. The in vitro and in vivo results obtained so far confirm this. A summary of the most important results is given in Chapter 14.2.2 in [13]. In addition, the use of magnetic hyperthermia in combination with other alternative therapies (double therapy) [13,16,17,18,19,20], or with the classic chemo- and radiotherapy [21,22,23], seem to be the most recent alternatives that have increased the effectiveness of cancer therapy, but also to reduced toxicity to healthy tissues. In this concept of double therapy, magnetic hyperthermia is used first, followed by chemo- and/or radiotherapy for the rest of the remaining tumor in order to reduce the toxicity on healthy tissues.

However, the success of superparamagnetic hyperthermia in cancer therapy depends, among other factors, fundamentally on the nanoparticles used for this therapy. Recent research in the field of magnetic hyperthermia aims to find the proper magnetic nanoparticles and the most suitable conditions and parameters to increase the efficacy on tumor destruction through this therapy (e.g., appropriate size of nanoparticles, optimum values of magnetic field amplitude and frequency, a certain packing fraction, dose and field exposure time, etc.) [3,24,25,26,27,28,29,30,31,32]. Some studies have shown that it is possible to deliver hyperthermal treatment with no off-target toxicity by the biocompatibility or biofunctionalization of magnetic nanoparticles using modern nanobiotechnology [33,34,35].

Bearing in mind our previous studies on superparamagnetic hyperthermia with CoFe_2_O_4_ nanoparticles [31] as well as the study on Fe_3_O_4_ nanoparticles decorated with γ-cyclodextrins [34,35], we propose through this study the use of a new core-shell structure of CoFe_2_O_4_ nanoparticles covered with γ-cyclodextrins (CoFe_2_O_4_–γ-CDs) as nanoheaters in superparamagnetic hyperthermia. From our study in [31] it was found that the CoFe_2_O_4_ nanoparticles could be used successfully in magnetic hyperthermia, having the major advantage of small nanoparticle size (~6 nm) compared to other nanoparticles [14,36] or, for example, with Fe_3_O_4_ nanoparticles where the optimum diameter is ~16 nm [5]. The use of cyclodextrins has multiple advantages for magnetic hyperthermia, primarily non-toxicity [34,37], which is a fundamental aspect compared to conventional chemo- and radiotherapy that have a high degree of toxicity on the body. Biocompatible polyacrylic acid (PAA) was used to bind γ-CDs to nanoparticles, which is suitable for this purpose [34]. We selected the use of γ-cyclodextrins for the coating of nanoparticles because these biostructures are the most stable compared to the smaller biostructures of α- or β-cyclodextrins [34]. The efficiency (i) of superparamagnetic hyperthermia can be increased by increasing the concentration of nanoparticles in suspension: nanoparticles being small, in the same volume, their number can be greatly increased (see Section 3.1). Increasing the effectiveness (ii) in destroying tumors is possible by performing intracellular therapy [38] in the case of smaller nanoparticles, where tumor cells are more efficiently destroyed from the inside. The elimination of toxicity (iii) is possible through the use of γ-CDs, which are perfectly biocompatible and non-toxic organic biostructures [34], currently used in the pharmaceutical and food industry. In conclusion, we consider this combination of small nanoparticles and biocompatibility with cyclodextrins as a very good strategy for:

- increasing the efficiency;
- increasing the efficacy in superparamagnetic hyperthermia to destroy tumors; and
- minimizing or even eliminating cytotoxicity.

These characteristics make them better for magnetic hyperthermia.

Considering the above aspects, in this paper, we studied by computer simulation the specific loss power generated in superparamagnetic hyperthermia using CoFe_2_O_4_ nanoparticles coated with γ-CDs (CoFe_2_O_4_–γ-CDs core-shell nanoparticles), and found the conditions under which the specific loss power becomes maximal in this case, depending on the size of the nanoparticles and the thickness of the biocompatible layer on the surface of the nanoparticles. The influence of the amplitude and frequency of the alternating magnetic field and the packing fraction of the nanoparticles on the maximum specific loss power was also studied. Moreover, we explained the dominant contribution of Brown relaxation processes to the maximum specific loss power as well as the unusual shifting of the maximum loss power to the higher diameter values in the presence of the γ-CDs layer on the nanoparticle surface.

## 2. Theoretical Considerations Regarding the Calculation of the Specific Loss Power in Superparamagnetic Hyperthermia with Core-Shell Nanoparticles Dispersed in a Liquid

In superparamagnetic hyperthermia, the specific loss power [14] for immobilized nanoparticles can be derived from the classic Debye model as [5]

(1) $$P_{s}=\frac{3\pi \mu_{0}\chi_{i}}{\rho \xi }\left(\coth\xi -\frac{1}{\xi }\right)\frac{2\pi f\tau_{N}}{1+{\left(2\pi f\tau_{N}\right)}^{2}}fH^{2}\;\left(\frac{W}{g}\right)$$

where $\chi_{i}$ is the initial magnetic susceptibility,

(2) $$\chi_{i}=\frac{\varepsilon \pi \mu_{0}M_{s}^{2}D^{3}}{18k_{B}T}$$

and ξ is the argument of the Langevin function,

(3) $$\xi =\frac{\pi \mu_{0}M_{s}D^{3}}{6k_{B}T}H$$

for the case of magnetization (*M*) of superparamagnetic nanoparticles [39,40],

(4) $$M=M_{sat}\left(\coth\xi -\frac{1}{\xi }\right)$$

and $\tau_{N}$ is the Néel magnetic relaxation time [41,42,43],

(5) $$\tau_{N}=\tau_{0}\cdot exp\left(\frac{\pi KD^{3}}{6k_{B}T}\right)$$

Other quantities in the above equations are: *H* is the amplitude of the alternating magnetic field; *f* is the frequency of the magnetic field; $\mu_{0}$ is the magnetic permeability of the vacuum (4π × 10^−7^ H/m); *ρ* is the density of the nanoparticle material; *ε* is the packing fraction of nanoparticle; *M_s_* is the spontaneous magnetization of nanoparticles; *D* is the diameter of nanoparticles considered spherical; *k_B_* is Boltzmann’s constant; *T* is room temperature; *M_sat_* is the saturation magnetization; $\tau_{0}$ is a time constant that is usually 10^−9^ s [44]; and *K* is the magnetic anisotropy constant.

However, when nanoparticles are not fixed and are dispersed in a pharmaceutical liquid, the contribution of relaxation due to brownian motion (by through the relaxation time) must be considered [42]:

(6) $$\tau_{B\;}=\frac{3\pi \eta D^{3}}{6k_{B}T}$$

In this equation, *η* is the viscosity coefficient of the dispersion liquid. In this case, the total relaxation time (*τ*) will be expressed by the equation [45]

(7) $$\frac{1}{\tau }=\frac{1}{\tau_{N}}+\frac{1}{\tau_{B}}$$

and may have different values depending on the ratio in which the two relaxation times Néel and Brown will be found.

Thus, under these conditions, the specific loss power will have the expression [35]

(8) $$P_{s}=\frac{3\pi \mu_{0}\chi_{i}}{\rho \xi }\left(\coth\xi -\frac{1}{\xi }\right)\frac{2\pi f\frac{\tau_{N\;}\tau_{B\;}}{\tau_{N\;}+\tau_{B\;}}}{1+{\left(2\pi f\frac{\tau_{N\;}\tau_{B\;}}{\tau_{N\;}+\tau_{B\;}}\right)}^{2}}fH^{2}\;\left(W/g\right)$$

This equation, together with all the above, will be used in our study for the 3D calculation of the specific power dissipated in superparamagnetic hyperthermia in the case of CoFe_2_O_4_ nanoparticles coated with γ-CDs and dispersed in saline.

## 3. Results and Discussion

### 3.1. Ferrimagnetic CoFe_2_O_4_ Nanoparticles Covered with γ-Cyclodextrins for Superparamagnetic Hyperthermia

**Figure 1:**
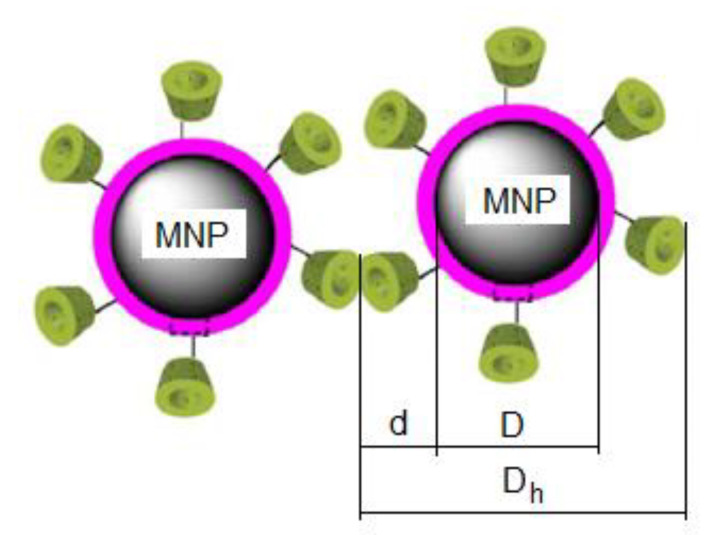
Schematic representation of magnetic nanoparticles coated with cyclodextrins (adapted from [53], © 2022 Elsevier B.V., with permission from Elsevier).

Our study on the specific loss power in superparamagnetic hyperthermia was conducted using *γ*-cyclodextrin-coated CoFe_2_O_4_ nanoparticles (CoFe_2_O_4_–γ-CDs) through PAA in a core-shell structure as in Figure 1, and dispersed in saline, in order to be used with increased efficiency and effectiveness in vitro, in vivo, and clinical trials in future.

The characteristic observables of CoFe_2_O_4_ nanoparticles, γ-CDs, and the alternating magnetic field used in our study are shown in Table 1. Due to the height of torus of ~8° A for γ-CDs [46] and binding polymer (PAA) of ~0.8 nm at the surface of the nanoparticles (MNP), we took into consideration the thickness of the organic layer (d) of 1.6 nm (Figure 1). For the thickness of the PAA layer, we took into account our results obtained by DLS and TEM in the case of Fe_3_O_4_-PAA–γ-CDs nanoparticles [47]. Bearing in mind our previous work [13,31,35] for parameters of magnetic field (*H*, *f*) and nanoparticle diameters (*D*), we considered for analysis the ranges in the table that are used in magnetic hyperthermia. Furthermore, for the packing fraction (*ε*), we considered the usual value in the table.

The use of nanoparticles of the CoFe_2_O_4_-γ-CDs core-shell combines the major advantages of using (i) small nanoparticles of CoFe_2_O_4_ for intracellular therapy (which is more effective in destroying tumors); (ii) non-toxicity of γ-CDs (very important aspect in current cancer therapy); and (iii) the very low thickness of the organic layer on the surface of nanoparticles (Figure 1). The latter aspect leads to the possibility of a high increase in the packing fraction of magnetic nanoparticles in suspension (increasing the concentration of magnetic nanoparticles (nanoheaters) in the same volume of dispersion liquid). As a result, there will be an increase in the specific loss power in this case, and implicitly in the heating temperature of the nanoparticles compared to other large biostructures used in magnetic hyperthermia (e.g., liposomes or different polymers) [26,49,50,51,52], where the increase in nanoparticle concentration is limited to a reduced value, with significant reduction in the efficient heating of nanoparticles, and negative effects in the magnetic hyperthermia of tumors (partial or inefficient destruction of tumors).

In addition, the use of γ-CDs to coat nanoparticles [53] also leads to the isolation of nanoparticles from each other (Figure 1), with multiple benefits in magnetic hyperthermia including the elimination of magnetic dipolar and van der Waals interactions between nanoparticles [54,55], obtaining of stable nanoparticle suspensions in time, non-reduction of loss power and heating temperature of nanoparticles due to interactions, and the formation of large agglomerates of nanoparticles, etc.

Bearing in mind the thickness *d* of the organic layer (γ-CDs and polyacrylic acid for binding it to nanoparticles) [35] from the surface of CoFe_2_O_4_ nanoparticles dispersed in the saline, in Equation (6), and then in Equations (7) and (8) will be considered in the calculations of the hydrodynamic diameter (*D_h_*) of the bionanoparticle CoFe_2_O_4_-γ-CDs (Figure 1):

(9) $$D_{h}=D+2d$$

instead of the diameter *D*. At the same time, in Equations (2), (3), (5) and (8), the diameter of the nanoparticle core *D* (sometimes called magnetic diameter) will be considered (Figure 1).

Thus, in this case, the Brown relaxation time will increase due to the increase in the physical diameter of the CoFe_2_O_4_ nanoparticle (from *D* to *D_h_*) by coating the nanoparticles with the biocompatible layer of γ-CDs.

### 3.2. Specific Loss Power in Superparamagnetic Hyperthermia with CoFe_2_O_4_–γ-CDs Nanoparticles

Using Equation (8) and Equations (2), (3), (5)–(7) and (9), and the data in Table 1, the specific loss power in superparamagnic hyperthermia with CoFe_2_O_4_ nanoparticles coated with γ-CDs and dispersed in a pharmaceutical liquid (saline) was calculated.

**Figure 2:**
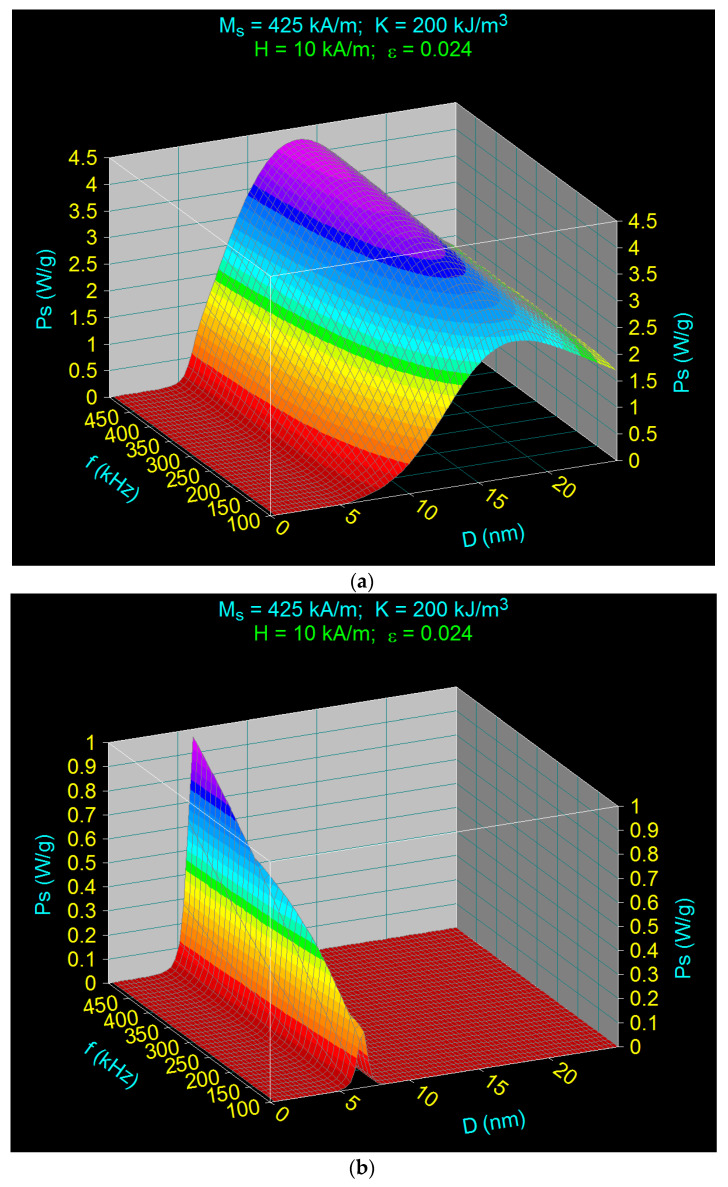

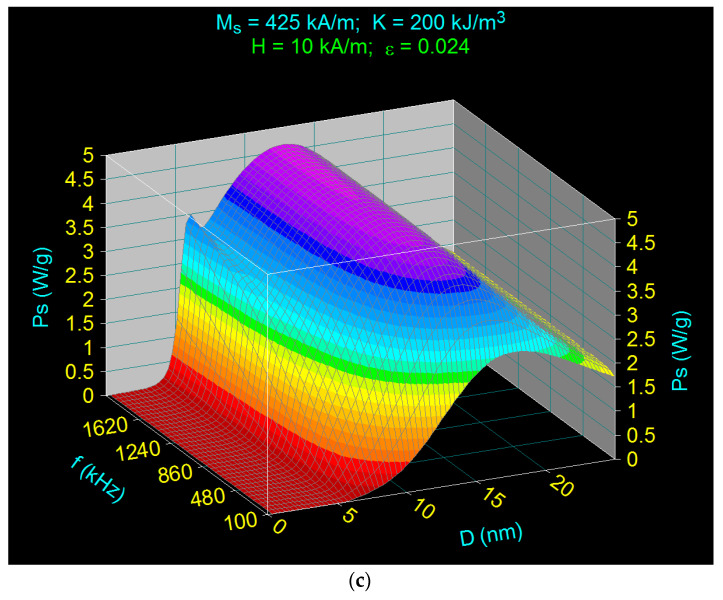
The specific loss power as a function of the nanoparticle diameter and the frequency of the magnetic field with amplitude of 10 kA/m, in the case of (**a**) nanoparticles of CoFe_2_O_4_–γ-CDs dispersed in saline, (**b**) immobilized nanoparticles of CoFe_2_O_4_, and (**c**) in the same condition as in case (**a**) but for extended frequency range up to 2000 kHz.

The result obtained in 3D, which is dependent on the size of the nanoparticles (diameter *D*) and the frequency of the alternating magnetic field (*f*), for the amplitude (*H*) of the magnetic field of 10 kA/m, is shown in Figure 2a.

At the same time, the specific loss power was calculated under the same conditions but in the absence of the γ-CDs layer from the surface of the CoFe_2_O_4_ nanoparticles and in the absence of the dispersion medium, the nanoparticles being considered immobile. For this, Equation (1) and Equations (2), (3) and (5) were used. The result obtained in this case is shown in Figure 2b.

In order to clearly see the contribution of Néel and Brown relaxation to the specific loss power, Figure 2c shows the power *P*_s_ in the case of Figure 2a but for a wide frequency range. Thus, in Figure 2c, the presence of two maxima of specific loss power can be clearly seen at a frequency of 2000 kHz. There is a specific value at which a second local maximum emerges.

Such a variation with two maxima of specific loss power was also observed by Fortin et al. [56] in the case of water-dispersed CoFe_2_O_4_ nanoparticles, for a frequency of 1 MHz and a magnetic field amplitude of 24.8 kA/m. A similar result was obtained by Zhang et al. [57] for cubic-shaped CoFe_2_O_4_ nanoparticles coated with coffeic acid (CFNPs) and dispersed in water, at a certain coating thickness and for a frequency of 115 kHz and a magnetic field of 27 kA/m.

The variation of the specific loss power according to the nanoparticle size and frequency followed the general trend obtained experimentally in the case of CoFe_2_O_4_ nanoparticles dispersed in aqueous solvents [56,57]. However, there were quantitative differences between our theoretical and experimental results, which can be explained by the different sizes of the parameters considered in the experiment (e.g., magnetic field amplitude, nanoparticle size, size distribution, packing fraction, magnetic anisotropy constant, medium viscosity). At the same time, our results were in agreement with other theoretical [14,36,58] and experimental [57,59] studies for CoFe_2_O_4_ nanoparticles [14,57] and other nanoparticle systems [36,58,59].

In both cases, the diameter (*D_M_*) at which the maximum specific loss power (*P_sM_*) occurs also moved to smaller diameter sizes (*D*) when the frequency (*f*) increased, as is known in general in magnetic hyperthermia [14,36,57]. Moreover, the value of the diameter of the CoFe_2_O_4_ nanoparticles at which the maximum loss power was obtained (Figure 2b) is in agreement with the one obtained in [14].

However, in the case of CoFe_2_O_4_ nanoparticles coated with γ-cyclodextrins (CoFe_2_O_4_–γ-CDs) (Figure 2a,c), the following results are different:

- the maximum specific loss power was significantly higher (approx. 4 times) than in the case of nanoparticles without γ-CDs (CoFe_2_O_4_) (Figure 2b); this issue was also observed by Fortin et al. [56] for water-dispersed CoFe_2_O_4_ nanoparticles for the field of 24.8 kA/m and under 500 kHz frequency; also, Zhang et al. [57] found the same trend in the case of cubic CoFe_2_O_4_ nanoparticles coated with caffeic acid and dispersed in water for a frequency of 115 kHz and a field of 27 kA/m;
- moreover, the shift of the maxima of the specific loss power at lower values of the nanoparticle diameters when the frequency increased was more accentuated in the case of CoFe_2_O_4_-γ-CDs nanoparticles compared to uncovered immobile CoFe_2_O_4_ nanoparticles;
- additionally, the values of the diameters at which the maximum specific loss power was obtained in the case of CoFe_2_O_4_–γ-CDs nanoparticles were higher (~13–17 nm, depending on the frequency) than in the case of the CoFe2O4 nanoparticles (~6–6.5 nm, depending on the frequency); this result is in agreement with the experimental result obtained in [57] regarding the variation with maximum of the specific loss power for the size of nanoparticles in the range of ~10–13 nm, in the case of cubic CoFe_2_O_4_ nanoparticles coated with hydrophilic caffeic acid molecules of 0.87 nm in thickness and dispersed in water, for 115 kHz and different amplitudes of the magnetic field (12–30 kA/m); the smaller value in the size of the nanoparticles diameter in the experiment would be due to the cubic shape of the nanoparticles used [57] compared to our nanoparticles, which were approximately spherical;
- the diameter of the nanoparticles in the case of nanoparticles of CoFe_2_O_4_-γ-CDs was no longer such a critical parameter as in the case of CoFe_2_O_4_ nanoparticles, as the maximum specific loss power in the first case was quite wide compared to the other case where the maximums were very narrow; and
- the increase in maximum specific loss power with increasing frequency of the alternating magnetic field in the case of CoFe_2_O_4_-γ-CDs nanoparticles was significantly lower than in the case of CoFe_2_O_4_ nanoparticles.

In conclusion, from the point of view of magnetic hyperthermia, we can say that the use of CoFe_2_O_4_-γ-CDs nanoparticles leads to the following major advantages for magnetic hyperthermia:

- high increase in maximum specific loss power, with a direct effect on the effectiveness of the method in tumor therapy;
- nanoparticle size is no longer as critical as CoFe_2_O_4_ nanoparticles, which is a practical advantage: nanoparticles with a wider diameter distribution can be used, which are generally obtained by preparation methods (obtaining nanoparticles with a very narrow distribution is difficult to achieve in practice); and
- the slower increase in specific loss power with increasing frequency in the case of CoFe_2_O_4_-γ-CDs nanoparticles is another advantage in magnetic hyperthermia because lower frequency magnetic fields can be used without losing much power compared to the case of nanoparticles not covered with γ-CDs, where the loss of power is accentuated.

In addition, the use of nanoparticles of CoFe_2_O_4_-γ-CDs in magnetic hyperthermia leads to the biocompatibility of nanoparticles with the biological environment, and to the elimination of cellular toxicity, as γ-CDs are perfectly biocompatible oligosaccharide organic structures. Moreover, the use of CoFe_2_O_4_-γ-CDs nanoparticles also leads to the elimination of magnetic interactions between nanoparticles and van der Waals (see Section 3.1), which would lead to the agglomeration of nanoparticles, with negative effects on magnetic hyperthermia such as decreased specific loss power, increased cellular toxicity in the case of large agglomerates of nanoparticles, sedimentation of nanoparticles in the case of agglomerates, suspensions unstable in time, etc.

The variation in the specific loss power in Figure 2a for the case of CoFe_2_O_4_-γ-CDs nanoparticles is due to Brown relaxation (rotation of nanoparticles in liquid simultaneously with the magnetic moment of nanoparticles) compared to the CoFe_2_O_4_ nanoparticles (Figure 2b), where the specific loss power is determined exclusively by the Néel relaxation processes (rotation of magnetic moments in immobile nanoparticles). This is clear from the diagram in Figure 2c, where the results show that for frequencies above 1500 kHz, the two contributions are clearly visible: the narrow maximum from small diameters (and high frequencies) given by Néel relaxation, and the wide maximum from diameters significantly higher, given by Brown relaxation.

In the case of our study, and in general, having the view that in magnetic hyperthermia, the frequencies higher than 1000 kHz are not used, the contribution of Néel relaxation processes to the maximum specific loss power in the case of CoFe_2_O_4_-γ-CD nanoparticles is practically negligible. This aspect is very important, and is another advantage in superparamagnetic hyperthermia, because in the case of CoFe_2_O_4_-γ-CD nanoparticles, where the specific loss power and the heating of the nanoparticles occurs exclusively through Brown relaxation processes and not Néel or Néel–Brown, larger magnetic fields can be used without the need to ensure the linearity of the magnetization for small magnetic fields, as in the case of Néel relaxation [5].

The result obtained in the case of CoFe_2_O_4_-γ-CD nanoparticles dispersed in saline is totally different from that obtained in the case of Fe_3_O_4_-γ-CDs nanoparticles [35], where the contribution of Néel relaxation processes is dominant in magnetic hyperthermia and not Brown. Moreover, the maximum specific loss power in the case of CoFe_2_O_4_-γ-CDs nanoparticles was obtained at significantly smaller nanoparticle diameters (e.g., ~13 nm compared to ~16 nm for Fe_3_O_4_–γ-CDs nanoparticles, at the 10 kA/m field, and the 500 kHz frequency).

In conclusion, we can say that in the case of nanoparticles of CoFe_2_O_4_-γ-CDs dispersed in saline, the specific loss power is obtained exclusively by Brown relaxation processes, having the specific characteristics presented above. This aspect is demonstrated more clearly in the next section.

### 3.3. Néel and Brown Magnetic Relaxations in CoFe_2_O_4_ Nanoparticles Covered with γ-CDs

**Figure 3:**
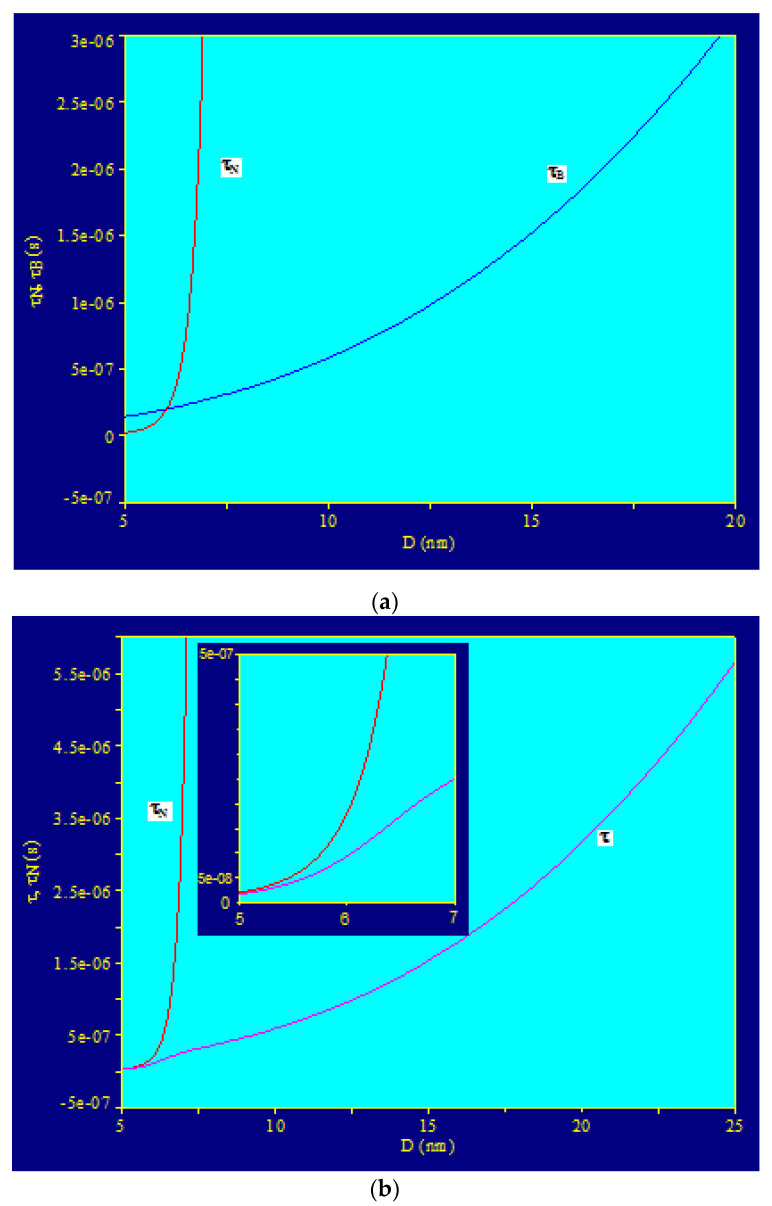
Relaxation times depending on the diameter of CoFe_2_O_4_ nanoparticles coated with γ-CDs and dispersed in saline: (**a**) Néel relaxation time and Brown relaxation time; (**b**) Néel relaxation time and total relaxation time (inset: enlarged image in the range where $\tau_{N}$ and τ become comparable in value).

Representing the variation of Néel ($\tau_{N}$), Brown ($\tau_{B}$), and total relaxation times (*τ*) in the case of CoFe_2_O_4_ nanoparticles coated with liquid-dispersed γ-CDs, depending on the nanoparticle diameter (Equations (5)–(7), with Equation (9)), the curves in Figure 3 were obtained. The obtained results clearly showed that the Néel relaxation contribution was obtained at small nanoparticle sizes, for diameters <~6 nm, where the Néel relaxation time was significantly shorter than the Brown relaxation time (Figure 3a,b), and at large sizes of nanoparticles of >~7 nm, the contribution of relaxation was the Brown type, where the Brown relaxation time was significantly shorter than the Néel relaxation time. Therefore, at a larger nanoparticle diameter, the total relaxation time (Equation (7)) is determined only by the Brown relaxation time (Figure 3b). In the range of values of diameters of approx. 5.7–6.4 nm, there was a contribution of both magnetic relaxation processes in certain proportions (Figure 3b, inset), and at approx. 6.1 nm, their contributions became equal (the two relaxation times became equal, $\tau_{N}$ = $\tau_{B}$).

These variations in the relaxation times obtained in the case of nanoparticles of Fe_3_O_4_-γ-CDs dispersed in saline explain why the specific power dissipated in magnetic hyperthermia changes, as shown in Figure 2a,c.

In conclusion, in the case of magnetic hyperthermia with liquid-dispersed CoFe_2_O_4_-γ-CDs nanoparticles, only Brown relaxation processes will contribute to the specific loss power in the usual frequency range for magnetic hyperthermia (up to 1000 kHz). This behavior is due to the high magnetic anisotropy of the CoFe_2_O_4_ nanoparticles, which causes the Néel relaxation time to increase a lot, even for small sizes of nanoparticles (over approx. 6.5–7 nm).

The results obtained in the case of liquid-dispersed CoFe_2_O_4_-γ-CDs nanoparticles differ greatly from those obtained in the case of Fe_3_O_4_-γ-CDs nanoparticles [35]. This is due to the high difference in the magnetic anisotropy of the two types of nanoparticles, with the magnetic anisotropy being much smaller in the case of Fe_3_O_4_ (magnetite) nanoparticles [48].

### 3.4. Effect of Magnetic Field Amplitude on Maximum Specific Loss Power in the Case of CoFe_2_O_4_-γ-CDs Nanoparticles

**Figure 4:**
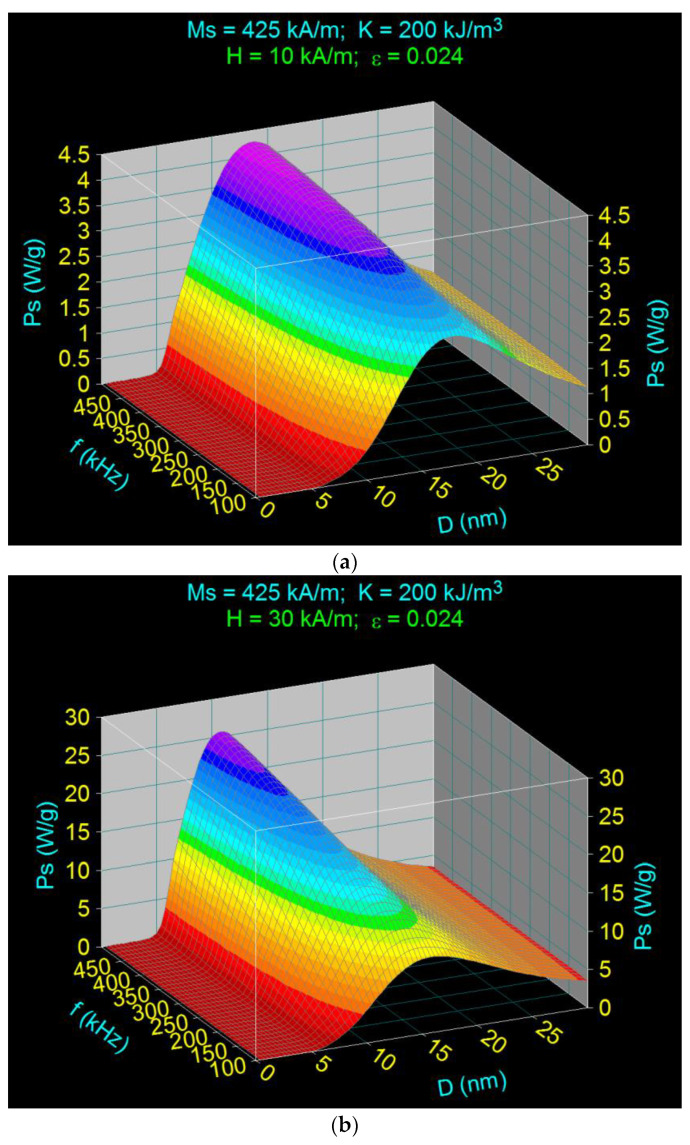

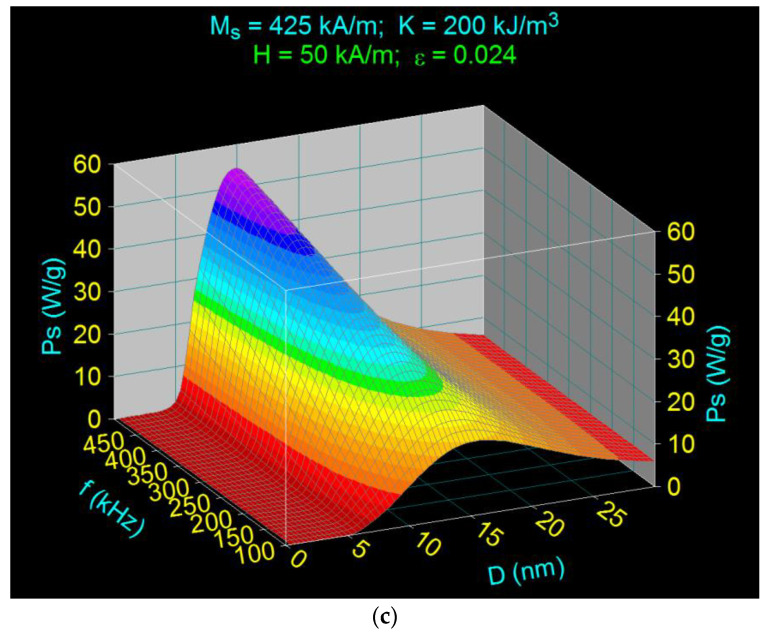
Variation of specific loss power in the case of nanoparticles of CoFe_2_O_4_-γ-CDs dispersed in saline, depending on the nanoparticle diameter and frequency, for different amplitudes of the magnetic field: (**a**) 10 kA/m, (**b**) 30 kA/m, and (**c**) 50 kA/m.

Applying an increasing magnetic field up to 50 kA/m, a radical change in the specific loss power and the shape of its maximum is obtained, an effect that is more pronounced at higher frequencies toward 500 kHz (Figure 4). When the amplitude of the magnetic field increases, a significant shift of the maximum specific loss power to smaller values of the nanoparticle diameters can be observed (e.g., to increase the magnetic field from 10 kA/m to 50 kA/m, the maximum specific loss power shifts from a diameter of 13.6 nm to a new position corresponding to a diameter of 9.8 nm, for the same frequency of 500 kHz).

The nanoparticle diameters (*D_M_*) corresponding to the maximum specific loss power (*P_sM_*) obtained for the frequencies of 500 kHz and 100 kHz are shown in Table 2 and Table 3. From these data, it can be observed that the effect of increasing the magnetic field on the displacement of the maximum specific loss power is more accentuated at the frequency of 500 kHz compared to 100 kHz.

**Figure 5:**
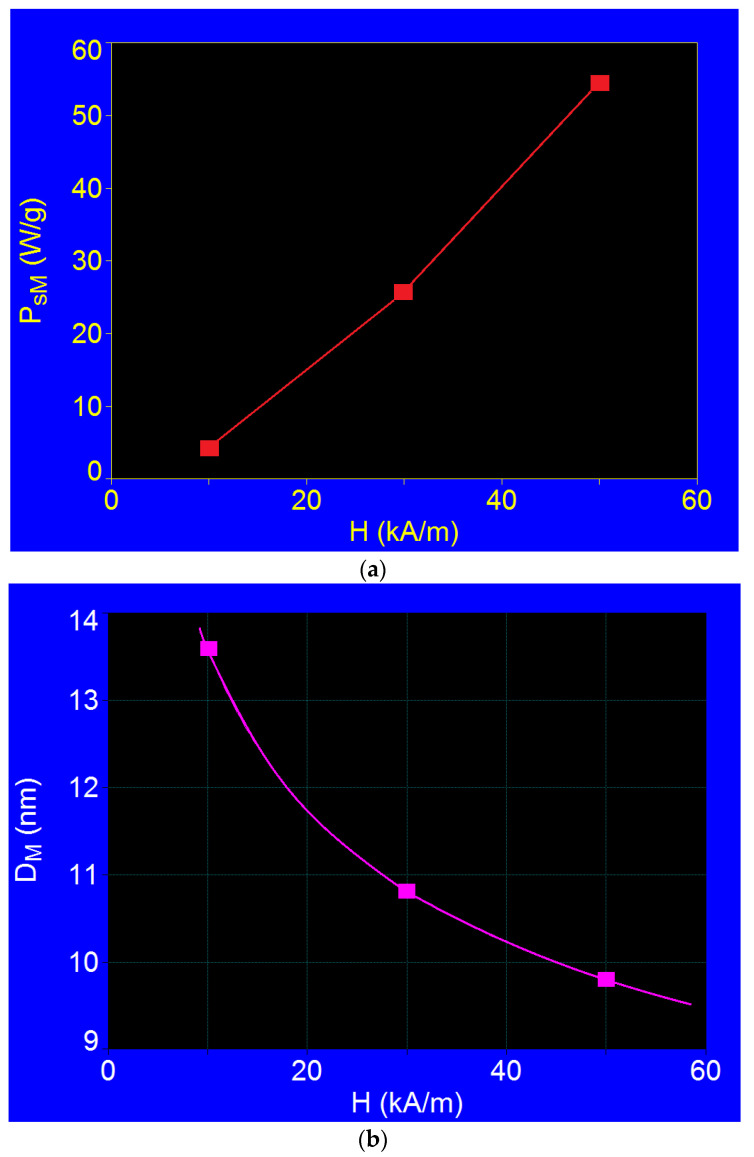
(**a**) Maximum specific loss power variation, and (**b**) nanoparticle diameter corresponding to maximum power, depending on the amplitude of the magnetic field at 500 kHz.

The variations of the maximum specific loss power (*P_sM_*) and the nanoparticle diameter corresponding to the maximums (*D_M_*) depend on the amplitude of the magnetic field for the frequency of 500 kHz, which are shown in Figure 5a,b. It can be observed that, while the maximum power increased quickly and almost linearly with the increase in the magnetic field amplitude (in the magnetic field considered), the diameter of the nanoparticles had a more pronounced decrease up to 30 kA/m, followed by a slower decrease in the rest of the range. Moreover, although the size of the nanoparticles corresponding to the maximum power decreased significantly (Figure 5b), the maximum specific loss power did not decrease, but increased, which occurred even more quickly (Figure 5a).

Similar variations were obtained for the frequency of 100 kHz, the values of the maximum specific loss power still being significantly lower, and the values of the diameter corresponding to the maximums of the power were significantly higher (Table 3) (e.g., for the 30 kA/m field, the difference in diameter was 5.2 nm).

A similar increase in the specific loss power as a function of magnetic field was observed experimentally at 195 kHz for EDT-coated CoFe_2_O_4_ nanoparticles (EDT: N-(trimethoxysilylpropyl)-ethylenediaminetriacetate) of ~20 nm in diameter dispersed in deionized water and ethylene glycol for the concentration of 1 mg/mL and 2 mg/mL [60]. Additionally, Fortin et al. [56] showed, at 700 kHz, a similar increase in the specific loss power as a function of the magnetic field for CoFe_2_O_4_ nanoparticles of ~4 nm and ~9 nm dispersed in water, and for ~10 nm dispersed in a mixture of water and glycerol.

From the point of view of magnetic hyperthermia, the entire range for the chosen magnetic field is viable, and even more suitable for larger fields, where significantly higher powers are obtained. In addition, the decrease in nanoparticle diameter when the magnetic field amplitude increases is a major advantage in terms of magnetic hyperthermia, for at least three reasons:

- decreasing the size of nanoparticles allows their easier penetration into tumor cells, with a beneficial effect on increasing the effectiveness of magnetic hyperthermia by destroying tumor cells inside them (performing intracellular therapy);
- the size of the nanoparticles can be controlled by the applied field, which allows the optimization of the magnetic hyperthermia by using nanoparticles of different sizes; and
- reducing the size of nanoparticles will also help reduce any cellular toxicity that may exist after therapy.

Furthermore, as the frequency of the magnetic field increases from 100 kHz to 500 kHz, the effect already known in magnetic hyperthermia is also observed: the displacement of the maximum specific loss power to lower values of the diameters that give these maximums. However, this effect is much more pronounced in the case of liquid-dispersed CoFe_2_O_4_-γ-CDs nanoparticles than in the case of CoFe_2_O_4_ nanoparticles [31].

Another important aspect observed was the fact that at higher magnetic fields and frequencies (e.g., 50 kA/m and 500 kHz), the maximum of specific loss power was narrowed (Figure 4c), and the diameter of the nanoparticles became quite critical compared to the case of small fields (10 kA/m, 500 kHz) (Figure 4a) or small fields and frequencies (10 kA/m, 100 kHz) (Figure 4a), where the maximum power became very wide and the diameter was no longer a critical parameter.

### 3.5. Influence of Magnetic Field Frequency on Maximum of the Specific Loss Power in the Case of CoFe_2_O_4_-γ-CDs Nanoparticles Dispersed in Saline

**Figure 6:**
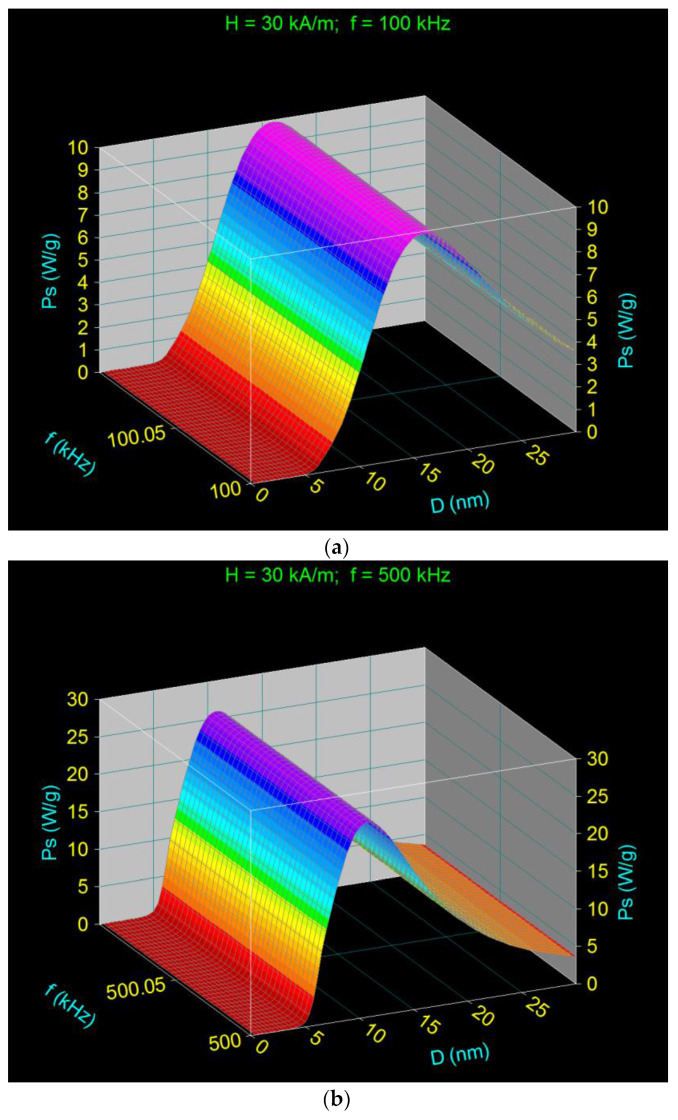

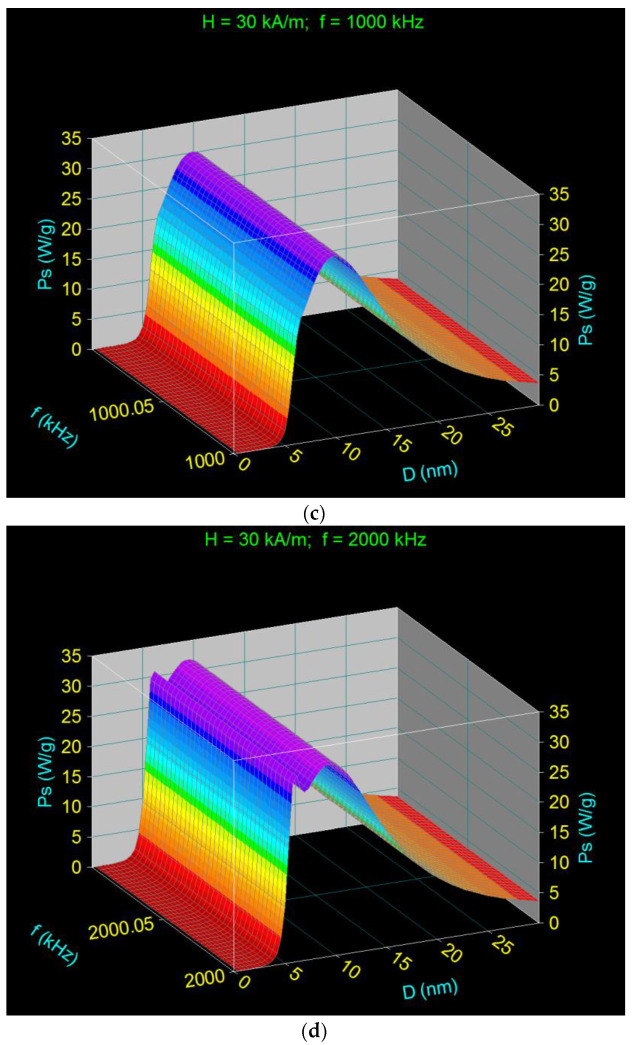
The variation of the specific loss power (*P_s_*) in the case of dispersion of CoFe_2_O_4_-γ-CDs nanoparticles, depending on the diameter of the nanoparticles, for the amplitude of the magnetic field of 30 kA/m and different frequencies: (**a**) 100 kHz, (**b**) 500 kHz, (**c**) 1000 kHz, (**d**) 2000 kHz.

The influence of the magnetic field frequency on the maximum specific loss power in CoFe_2_O_4_-γ-CDs nanoparticles was studied for the applied magnetic field of 30 kA/m by extending the frequency range up to 2000 kHz, in order to capture the effect of Néel relaxation. The results obtained are shown in Figure 6. Similar effects were observed for the other values of the magnetic field: 10 kA/m and 50 kA/m.

**Figure 7:**
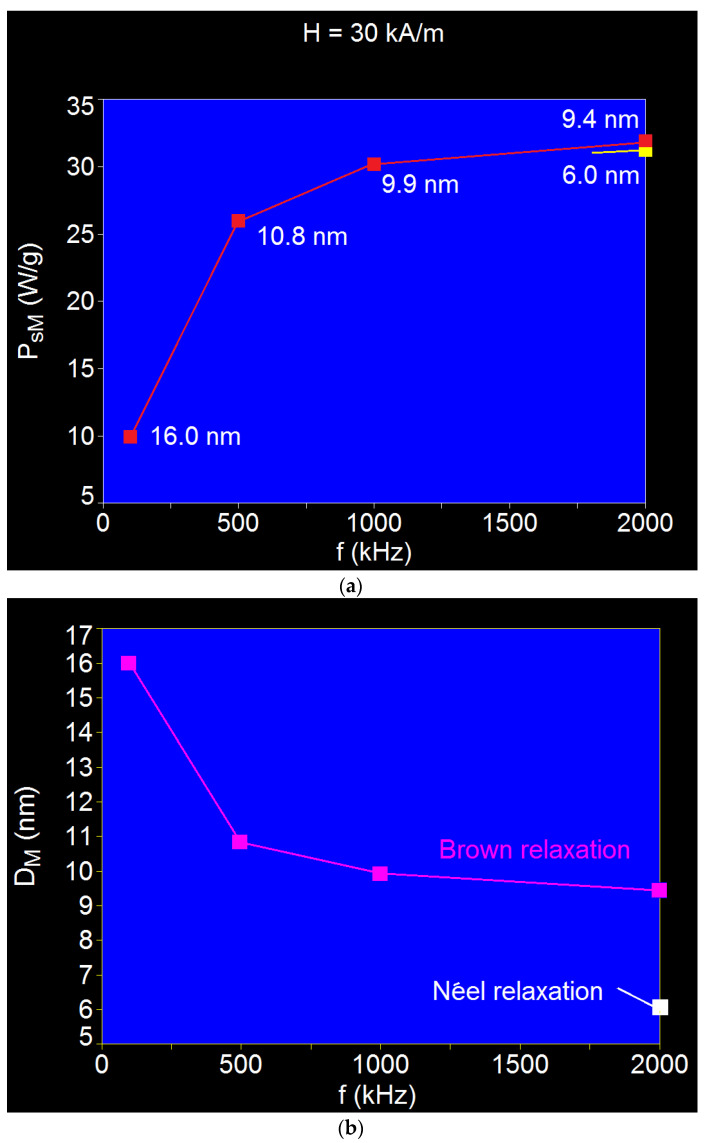
(**a**) Variation of the maximum specific loss power (*P_sM_*) and (**b**) nanoparticle diameter corresponding to the maximum (*D_M_*), depending on the frequency, for the magnetic field of 30 kA/m.

Increasing the frequency of the alternating magnetic field led to major changes in the spectrum of the specific loss power, both as the shape and type of magnetic relaxation (Figure 6), and as values (Table 4) and the shape of variation (Figure 7a). Additionally, the maximum specific loss power (*P_sM_*) was obtained at different values of the nanoparticle diameters (*D_M_*) for different frequencies (Table 4).

The results obtained show:

- fast increase in the maximum specific loss power (*P_sM_*) up to the frequency of ~500 kHz followed by a slow increase up to ~1000 kHz, and then a saturation effect up to 2000 kHz (Figure 7a); an almost linear increase in specific loss power with increasing frequency in the 300–1100 kHz range followed by a saturation of power at high frequencies was also observed in [56] depending on the size of the nanoparticles and for the 15.2 kA/m magnetic field;
- narrowing of the maximum specific loss power as the frequency increased (Figure 6);
- above the frequency of 1000 kHz, the Néel magnetic relaxation appeared (Figure 6c) in addition to the Brown relaxation, which gave a separate maximum of approx. the same power intensity at 2000 kHz (Figure 6d and Figure 7a (red and yellow points));
- although the maximums of the specific loss powers obtained at the frequency of 2000 kHz given by the Néel relaxation and the Brown relaxation were approximately equal, these were still obtained at totally different diameters: 9.4 nm for the maximum power given by the Brown relaxation, and 6.0 nm for the maximum power given by the relaxation Néel (Figure 6d and Table 4);
- the diameters of the nanoparticles at which the maximum specific loss power was obtained decreased with increasing frequency, namely, they decreased rapidly up to 500 kHz and then slowly up to 1000 kHz, followed by a saturation effect up to 2000 kHz (Figure 7b); and
- at frequencies around 1000 kHz, the effect of Néel relaxation on the maximum specific loss power was masked by the Brown relaxation, which was more pronounced and has a wide maximum (Figure 6c); therefore, the exact value of the Néel relaxation diameter at this frequency was difficult to determine (Figure 7b); however, at the frequency of 2000 kHz, the maximum specific loss power given by the Néel relaxation was clearly delimited (Figure 6d) as well as the value of its corresponding diameter (Figure 7b (yellow point)).

However, in light of the results shown above and those in Figure 7a, in terms of magnetic hyperthermia, the frequency range up to 1000 kHz is recommended, where Brown relaxation is the dominant process that contributes to the maximum specific loss power.

### 3.6. Increasing the Maximum Specific Loss Power in Magnetic Hyperthermia with CoFe_2_O_4_-γ-CDs Nanoparticles Dispersed in Saline

In practical applications, it is very important to apply magnetic hyperthermia within the specific conditions in which the highest specific loss power can be obtained, in order to increase the efficiency and effectiveness of the method in tumor therapy. Given this, below we will analyze the maximum specific loss power that could be obtained in the case of dispersed CoFe_2_O_4_-γ-CDs nanoparticles according to the possible basic parameters in magnetic hyperthermia: amplitude of the alternating magnetic field (*H*), frequency alternating magnetic field (*f*), nanoparticle size (diameter *D*), and packing fraction of nanoparticles. Taking into account the above results obtained for the CoFe_2_O_4_-γ-CDs nanoparticles, and those previously obtained for CoFe_2_O_4_ nanoparticles [31], we will consider in our calculation the values given in Table 5, the other quantities from Equation (8), and the Equations (2), (3), (5)–(7) and (9), and the data from Table 1, remaining unchanged.

**Figure 8:**
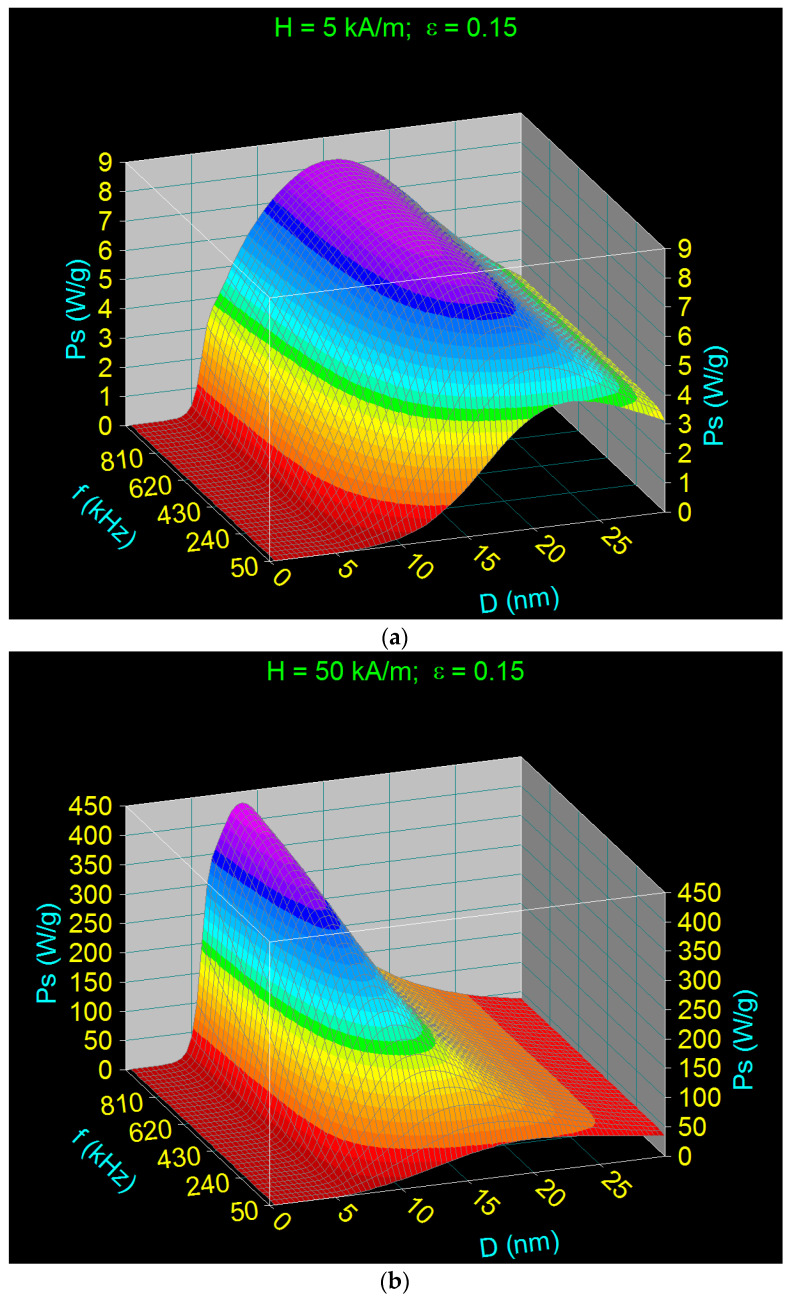

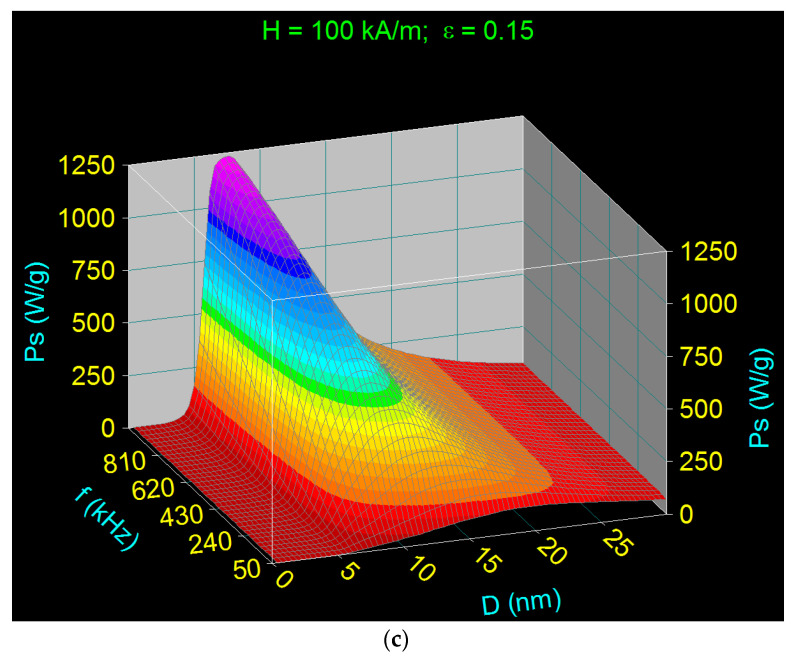
Variation of the specific loss power in the case of CoFe_2_O_4_-γ-CDs nanoparticles dispersed in saline, depending on the diameter of the nanoparticles in the frequency range 50–1000 kHz, for the packing fraction of 0.15, and different values of the magnetic field: (**a**) 5 kA/m, (**b**) 50 kA/m, (**c**) 100 kA/m.

The 3D variations of the specific loss power obtained, depending on the diameter of the nanoparticles and the frequency of the magnetic field, in this case, for three values of the amplitude of magnetic field (minimum value of 5 kA/m in the field, middle 50 kA/m, and maximum of 100 kA/m), and the packing fraction of 0.15 (Table 5), are shown in Figure 8.

The results obtained show that quite a high maximum specific loss power can be obtained in MHT using CoFe_2_O_4_-γ-CDs nanoparticles by increasing the packing fraction of nanoparticles (concentration), magnetic field amplitude, and/or frequency for different values of nanoparticle sizes. In this case, this power is obtained exclusively through the Brown relaxation processes; even at the frequency of 1000 kHz, the Néel relaxation is negligible. The specific loss power (*P_s_*) strongly depends on these parameters (*H, f, ε, D*), and the power increases fast with the increase in the packing fraction (from 0.024 previously to 0.15), the amplitude, and frequency of the magnetic field. It should be noted that the increase in maximum power with frequency was faster at higher magnetic fields (Figure 8b,c). However, at a low magnetic field of 5 kA/m (Figure 8a), the maximum power obtained was very low, even in high frequency conditions (e.g., 1000 kHz), so that the heating of nanoparticles in this case will be inefficient for magnetic hyperthermia.

Furthermore, the maximum specific loss power (*P_sM_*) was obtained at a certain value of the nanoparticle diameter (*D_M_*), a diameter that is dependent on both the amplitude of the magnetic field and the frequency, as follows: (i) as the amplitude of the magnetic field increases, the diameter of the nanoparticles at which the maximum power is obtained decreases; and (ii) as the frequency increases, the nanoparticle diameter decreases.

However, the nanoparticle diameter remains a critical parameter at higher frequencies (over 500 kHz) and at high magnetic fields (over 50 kA/m) (Figure 8b,c), for different frequencies and amplitudes of the magnetic field, which are shown in Table 6. The values obtained for the diameters of nanoparticles that give the maximums of the specific loss power, and their corresponding powers, for different frequencies and amplitudes of the magnetic field, are given in Table 6.

The high decrease in this case of the nanoparticle diameter in a very wide range of values, from 22.8 nm in the low field and frequency (5 kA/m, 50 kHz)) (Figure 8a), up to 7.5 nm in the high field and frequency (100 kA/m, 1000 kHz), are important advantages for magnetic hyperthermia as it (i) increases the efficiency and effectiveness of magnetic hyperthermia in tumor therapy by increasing the specific loss power, (ii) reduces cell toxicity by reducing the size of nanoparticles, and (iii) achieves intracellular hyperthermia at small nanoparticle sizes. Thus, it is possible to tune the parameters of alternating magnetic fields (*H* and *f*), in order to find the optimal conditions for the efficient achievement of magnetic hyperthermia, with maximum effectiveness on tumors.

Another very important aspect in terms of increasing the efficiency of magnetic hyperthermia in this case is that for the entire range of the values of the amplitude and frequency of magnetic field, magnetic hyperthermia occurs only through Brown relaxation processes (rotating nanoparticles in suspension) and not Néel (rotation of magnetic moments in nanoparticles) or Néel–Brown, due to the high magnetic anisotropy of these nanoparticles and their coating with the γ-CDs layer. Thus, for the entire range of values for the field and frequency, the diameter of the nanoparticles leading to the maximum specific loss power is greater (Table 6) than that in the case of magnetic hyperthermia obtained by Néel relaxation processes, which is around 6–6.5 nm [31]. Therefore, higher or much higher maximum specific loss powers can be obtained for the entire range of values (depending on the field and frequency) in the case of magnetic hyperthermia with CoFe_2_O_4_-γ-CDs than in the case of CoFe_2_O_4_ nanoparticles not coated with cyclodextrins, which is beneficial in terms of magnetic hyperthermia and the effectiveness of this therapy in destroying tumor cells.

However, a more detailed analysis of these aspects with the purpose of determining the optimal conditions in magnetic hyperthermia will be made in future work, bearing in mind the certain limitations [61] existing for in vitro and in vivo applications of this therapy.

## 4. Conclusions

The results obtained in this study show that nanoparticles of CoFe_2_O_4_-γ-CDs can be successfully used in superparamagnetic hyperthermia for alternative cancer therapy in order to increase the effectiveness of tumors and non-toxicity on healthy cells.

The specific loss power in magnetic hyperthermia with saline-dispersed CoFe_2_O_4_-γ-CDs nanoparticles is obtained exclusively by Brown relaxation processes (Section 3.2), which leads to, at least, the following benefits for magnetic hyperthermia:

- increasing the value of the optimal diameter (which gives the maximum specific loss power) in a wide range of values ~(9–14) nm (depending on the amplitude and frequency of the magnetic field), which is more suitable for magnetic hyperthermia compared to ~(6–6.5) nm in the case of Néel relaxation for CoFe_2_O_4_ nanoparticles, simultaneously with the significant increase in the maximum specific loss power (increase of efficiency and effectiveness in therapy);
- the nanoparticle diameter is no longer a critical parameter as in the case of Néel relaxation, with advantages in the practical implementation of magnetic hyperthermia (the use of nanoparticles with wide diameter distributions); and
- the possibility of using larger magnetic fields, not being limited to small fields as in the case of Néel relaxation (which practically leads to the possibility of obtaining significantly higher specific loss powers in magnetic hyperthermia, with beneficial effects in hyperthermia).

However, in this case, the maximum specific loss power is indicated to be obtained up to the maximum frequency of 1000 kHz, where a maximum power saturation effect occurs, optimal for the range of ~(200–500) kHz, depending mainly on the amplitude of the magnetic field.

Changing the amplitude of the magnetic field radically changes, in this case, the value of the optimal diameter (*D_M_*), which gives the maximum specific loss power in magnetic hyperthermia (*P_sM_*), in contrast to cases when the maximum power in magnetic hyperthermia is determined by Néel relaxation and the optimal diameter does not change.

The presence of γ-CDs on the surface of CoFe_2_O_4_ nanoparticles, in addition to ensuring biocompatibility and cellular non-toxicity in magnetic hyperthermia, and the elimination of interactions between magnetic nanoparticles, which are major benefits in magnetic hyperthermia, also allows for a significant increase in the packing fraction of nanoparticles (the concentration of nanoparticles). In this case, this leads to a significant increase in the specific loss power and heating temperature (efficiency in magnetic hyperthermia) (Section 3.6), thus allowing the use of small magnetic fields to obtain superparamagnetic hyperthermia, compared to the use of other larger biostructures, for example, liposomes (with a size of tens–hundreds of nm), which significantly decrease the efficiency of magnetic hyperthermia.

The obtained results allow for the practical implementation, in optimal conditions, of superparamagnetic hyperthermia using nanoparticles of CoFe_2_O_4_-γ-CDs in order to increase the efficiency and effectiveness in tumor therapy without toxicity.

## Figures and Tables

**Table 1:** Characteristic observables of CoFe_2_O_4_-γ-CDs nanoparticles and alternating magnetic field; *M*_s_ is the spontaneous magnetization of nanoparticles; *K* is the anisotropy constant of CoFe_2_O_4_; *ρ* is the density of the material of nanoparticles; *ε* is the magnetic packing fraction of nanoparticles; *η* is the viscosity coefficient of the liquid; *d* is the thickness of the organic layer (PAA-CDs) on the surface of CoFe_2_O_4_ nanoparticles; *D* is the diameter of monodispersed nanoparticles; *H* is the alternating magnetic field; and *f* is the frequency of magnetic field.

*M_s_* * (kA/m)	*K* * (kJ/m^3^)	*ρ* * (×10^3^ kg/m^3^)	*ε*	*η* ** (kg/m.s)	*d* ** (nm)	*D* (nm)	*H* (kA/m)	*f* (kHz)
425	200	5.29	0.024	7 × 10^−4^	1.6	1–25	10–50	100–500

* [48]; ** [35].

**Table 2:** The maximum values of the specific loss power (*P_sM_*) in the case of nanoparticles of CoFe_2_O_4_-γ-CDs dispersed in saline, and the diameters of the nanoparticles corresponding to these maximums (*D_M_*), for three values of the magnetic field and the frequency of 500 kHz.

	Observables	*H* (kA/m)	*P_sM_* (W/g)	*D_M_* (nm)	Relaxation Type
No.					
1		10	4.28	13.6	Brown relaxation
2		30	25.66	10.8	Brown relaxation
3		50	54.56	9.8	Brown relaxation

**Table 3:** The maximum values of the specific loss power (*P_sM_*) in the case of nanoparticles of CoFe_2_O_4_-γ-CDs dispersed in saline, and the diameters of the nanoparticles corresponding to these maximums (*D_M_*), for three values of the magnetic field and the frequency of 100 kHz.

	Observables	*H* (kA/m)	*P_sM_* (W/g)	*D_M_* (nm)	Relaxation Type
No.					
1		10	2.56	17.7	Brown relaxation
2		30	9.90	16.0	Brown relaxation
3		50	17.45	15.6	Brown relaxation

**Table 4:** The maximum values of the specific loss power (*P_sM_*) and the diameters of the corresponding nanoparticles (*D_M_*) for different frequencies and the amplitude of the magnetic field of 30 kA/m.

	Observables	*f* (kHz)	*P_sM_* (W/g)	*D_M_* (nm)	Relaxation Type
No.					
1		100	9.90	16.0	Brown relaxation
2		500	25.96	10.8	Brown relaxation
3		1000	30.16	9.9	Brown relaxation
4		2000 2000	31.89 31.22	9.4 6.0	Brown relaxation Néel relaxation

**Table 5:** Values of the parameters of alternating magnetic field and nanoparticle diameter, and packing fraction.

	Observable	*H* (kA/m)	*f* (kHz)	*D* (nm)	*ε*
	
Range values		5–100	50–1000	1–30	0.15

**Table 6:** Nanoparticle diameter (*D_M_*) values corresponding to the maximum specific loss power (*P_sM_*) for different amplitudes and frequencies of the magnetic field.

	H = 5 kA/m		H = 50 kA/m		H = 100 kA/m	
*f* (kHz)	*D_M_* (nm)	*P_sM_* (W/g)	*D_M_* (nm)	*P_sM_* (W/g)	*D_M_* (nm)	*P_sM_* (W/g)
50	22.8	4.16	20.0	57.22	19.8	116.66
500	16.1	8.04	9.8	344.18	8.8	871.74
1000	15.6	8.22	8.8	430.73	7.5	1235.69

## Data Availability

This is not applicable.
